# Oesophagectomy in a patient with azygos vein continuation of the inferior vena cava: report of a case

**DOI:** 10.1186/s12957-015-0625-3

**Published:** 2015-08-12

**Authors:** Yi Shen, Xiang Zhuang, Ping Xiao, Wei Dai, Qiang Li

**Affiliations:** Graduate School, Guangxi Medical University, 22# Shuang Yong Road, Nanning, 530021 China; Department of Thoracic Surgery, Sichuan Cancer Hospital, 55# Renmin South Road, Chengdu, 610041 China

**Keywords:** Azygos vein, Oesophagectomy, Veno-venous bypass

## Abstract

The azygos system of veins varies greatly in its mode of origin, but the variation in which the azygos vein is a continuation of the inferior vena cava (IVC) is rare. During an oesophagectomy, the azygos vein typically is transected as a requirement of the surgery. In this case, the enlarged azygos and its arch were a continuation of the IVC. During our procedure, we first established a bypass between the right femoral vein and the jugular vein in case of injury to the azygos vein, and we then performed a McKeown oesophagectomy without transecting the azygos vein. Our experience suggests that an oesophagectomy in cases with an azygos vein continuation of the IVC is feasible. An adequate medical examination and careful reading of the imaging is crucial for the safety of these surgical procedures. An appropriate surgical approach should be selected according to the location of the tumour, the size of the tumour and its anatomical features. The establishment of a veno-venous bypass and protection of the azygos arch in patients whose azygos vein is a continuation of IVC is necessary.

## Background

In normal human anatomy, the azygos vein drains blood from the oesophageal, mediastinal, intercostal, pericardial and bronchial veins, and the inferior vena cava (IVC) collects the blood from the lower limbs, pelvic area and abdomen. In an oesophageal surgery procedure, the azygos arch is transected conventionally to facilitate the resection of carcinomas, lymph node dissection and gastrooesophageal anastomosis.

We report a case of an oesophageal carcinoma with an azygos vein continuation of the IVC. In this case, the azygos vein required protection, and the surgical procedure necessitated a veno-venous bypass between the left femoral vein and the jugular vein to guarantee an adequate venous return in the event that the azygos vein was injured.

## Case presentation

**Fig. 1:**
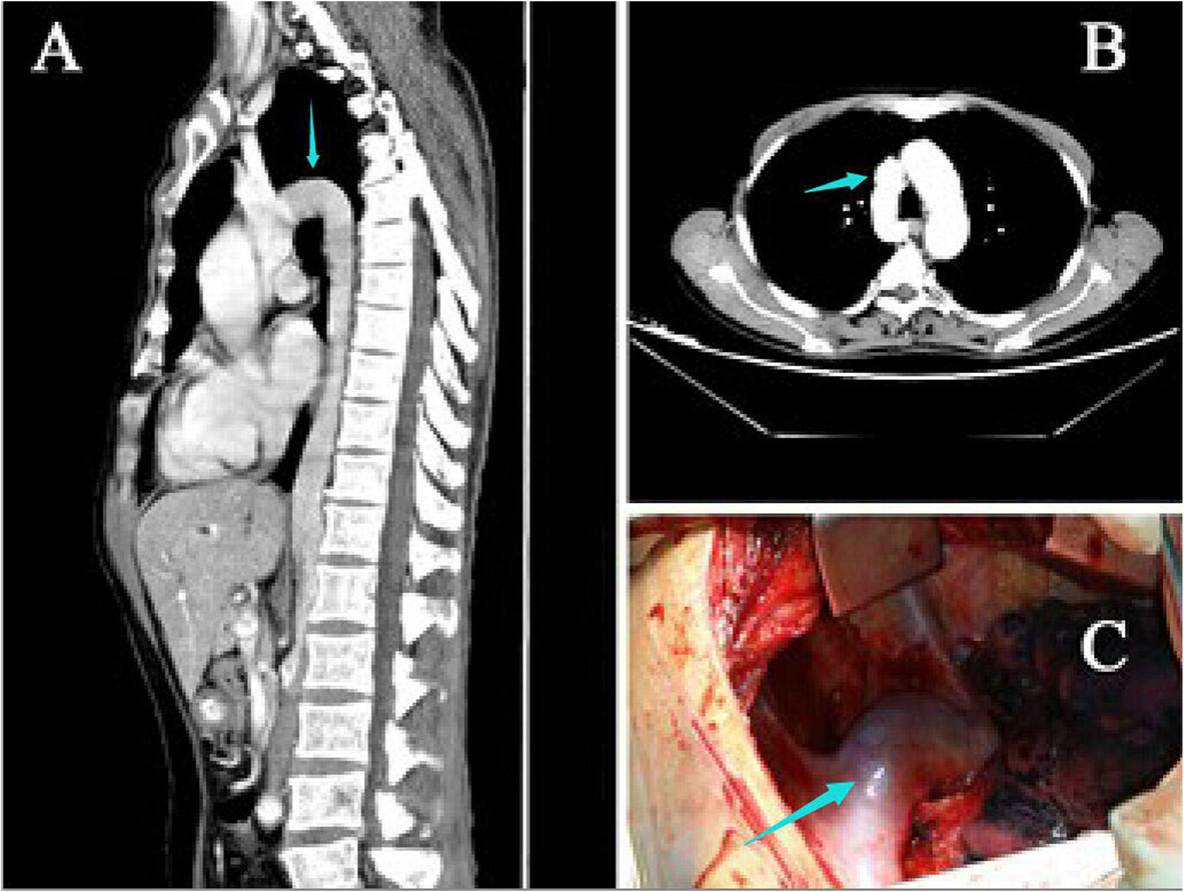
The enlarged azygos vein continuation of the inferior vena cava. **a** The azygos vein continuation of the IVC. **b** The CT scan revealed an enlarged arch of the azygos vein across the aortic arch. **c** Macroscopically, the diameter of the azygos vein was approximately 2.5 cm

A 56-year-old man presented with a 2-year history of dysphagia. He was diagnosed with a middle thoracic oesophageal squamous cell carcinoma by both a gastroscopy and a biopsy. A chest computed tomography (CT) scan showed an enlarged azygos vein (a diameter of 2.5 cm) that was a continuation of the IVC (Fig. 1a, b). A CT scan of the abdomen showed a defect in the suprarenal segment of the inferior vena cava and direct drainage of the hepatic vein into the right atrium.

After consultation with the thoracic surgery department clinicians and the anaesthesiology department, we decided to perform a McKeown oesophagectomy. The patient was anaesthetised with a double-lumen tube and underwent a standard posterolateral thoracotomy by traditional open surgery.

We first performed the jugular and femoral vein percutaneous puncture to create a veno-venous bypass and then connected the pressure sensor system to the monitor. The monitor displayed the real-time pressure value of the jugular vein and the femoral vein.

**Fig. 2:**
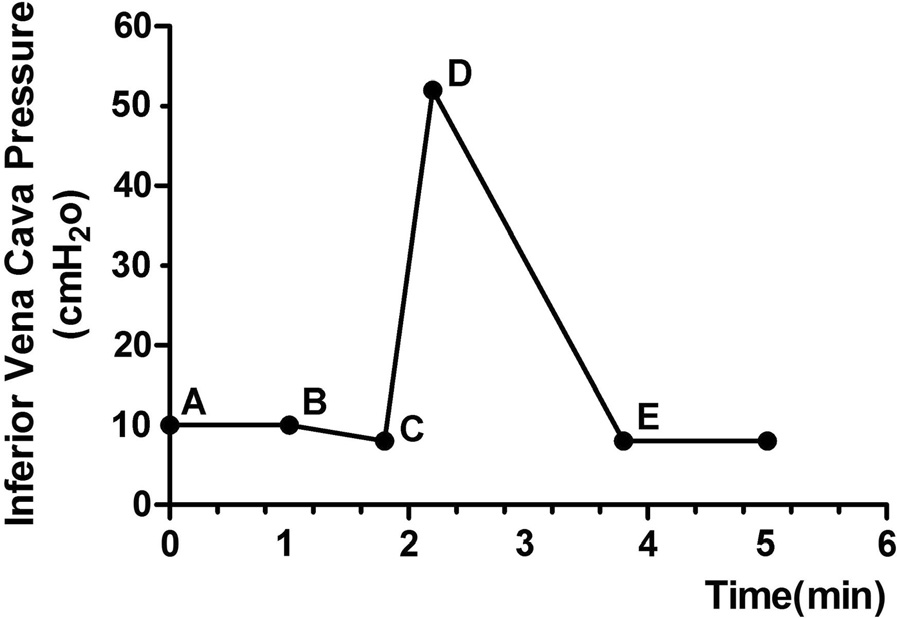
The traced waveform of the inferior vena cava pressure. *A*–*B*: The normal IVC pressure was approximately 10 mmHg. *B*–*C*: When the veno-venous bypass was open, the IVC pressure decreased to 8 cmH_2_O. *C*–*E*: When the azygos vein was temporarily blocked, the IVC pressure quickly increased to 52 cmH_2_O. When the azygos vein was opened, the IVC pressure gradually decreased to 8 cmH_2_O

Macroscopically, the diameter of the azygos arch was approximately 2.5 cm (Fig. 1c), and the superior edge of the tumour adhered tightly to the arch of the azygos vein. When the arch of the azygos vein was pulled with a rubber hose to dissociate it from the carcinoma, the monitor showed that the femoral vein pressure increased to 52 mmH_2_O; the pressure returned to a normal value when we opened the bypass between the femoral vein and the jugular vein (Fig. 2). The surgery was performed smoothly, without injury to the azygos vein, and the postoperative recovery was uneventful. The pathology of the resected specimen showed a poorly differentiated squamous cell carcinoma and no evidence of malignancy in 15 of the lymph nodes.

After 5 months of follow-up, the patient was asymptomatic, with no evidence of recurrent disease either clinically or on CT.

## Discussion

Cases of azygos vein enlargement have been reported frequently, but enlargement of the azygos vein with an azygos vein continuation of the IVC has an estimated prevalence of only 0.15 % [1]. Our patient had an asymptomatic congenital interruption of the IVC and no previous medical history. We discovered a tumour located behind the enlarged azygos arch, which was confirmed to be a continuation of the IVC on a chest CT scan. Conventionally, the azygos vein is transected to perform an oesophageal surgery. However, in this case, the azygos vein was enlarged due to an anomalous development of the IVC, and transection of the azygos vein would have resulted in venous hypertension and death [2]. Accordingly, an adequate medical examination and a careful reading of the imaging are crucial to ensure the safety of the surgical procedure.

This patient was diagnosed with a middle thoracic oesophageal squamous cell carcinoma by both a gastroscopy and a biopsy. We routinely perform radical resections of oesophageal carcinomas with an intrathoracic oesophagogastrostomy. In this case, the superior edge of the tumour was located at the back of the azygos arch and adhered tightly to it. The dilated gastric tube could have put pressure on the azygos vein and the azygos arch, which may have caused a blockage of the venous return from the lower limbs, pelvic area and abdomen. In addition, the enlarged azygos arch could have put pressure on the anastomotic stoma, which may have resulted in an anastomotic fistula. Therefore, a McKeown oesophagectomy was performed for this patient, and the gastric tube was pulled from the back of the sternum to the neck to perform the anastomosis.

Shintakuya et al. [3] have reported one case in which oesophagectomy was performed on a patient with a duplicated IVC, and azygos vein variation was identified by preoperative CT. Therefore, during oesophageal carcinoma surgery for patients with azygos vein variation, it is extremely important to maintain the integrity of the azygos vein. Preoperative thoracic and abdominal CT scans are also extremely important for oesophageal carcinoma patients. These tests are capable of not only helping to identify tumour stages and determine treatment methods but also revealing vascular and organ variations, allowing for the preparation of appropriate preoperative measures to ensure surgical safety.

The veno-venous bypass is rarely applied to oesophageal surgery. In this case, the enlarged azygos arch adhered tightly to the tumour, which meant that the azygos vein could have been injured during the oesophageal surgery procedure. Therefore, we first established an extracorporeal bypass between the femoral vein and the jugular vein to guarantee an adequate venous return in the event that the azygos vein was injured.

## Conclusions

We herein reported a rare case of an oesophagectomy with an azygos vein continuation of the IVC. We performed a McKeown oesophagectomy successfully without the transection of the azygos vein. Several important aspects to consider follow. First, an adequate medical examination and a careful reading of the images are crucial for the safety of this surgical procedure. Second, an appropriate surgical approach should be selected according to the location of the tumour, the size of the tumour and its anatomical features. Third, it is necessary to establish a veno-venous bypass and to protect the azygos arch in patients whose azygos vein is a continuation of the IVC.

## Consent

A written informed consent was obtained for publication of this report and accompanying images. A copy of the written consent is available for review by the Editor-in-Chief of this journal.
